# Higher Levels of Serum Leptin Are Linked with a Reduction in Gait Stability: A Sex-Based Association

**DOI:** 10.3390/biom15020195

**Published:** 2025-01-29

**Authors:** Mahmoud A. Alfaqih, Ebaa Ababneh, Yousef Khader, Khawla Mhedat, Mai Sater

**Affiliations:** 1 Department of Biochemistry, College of Medicine and Health Sciences, Arabian Gulf University, Manama 15503, Bahrain; maiss@agu.edu.bh; 2 Department of Physiology and Biochemistry, Faculty of Medicine, Jordan University of Science and Technology, Irbid 22110, Jordan; eyababneh@just.edu.jo (E.A.); kralmhidat10@sci.just.edu.jo (K.M.); 3 Department of Community Medicine, Public Health and Family Medicine, Faculty of Medicine, Jordan University of Science and Technology, Irbid 22110, Jordan; yskhader@just.edu.jo

**Keywords:** leptin, gait stability, physical activity, double support time, walking asymmetry

## Abstract

Gait stability prevents falls and injuries during physical activities. Muscle strength, aging, and co-existing chronic diseases are factors that affect gait stability. Leptin is an adipokine with pro-inflammatory properties. Several reports demonstrated an association between serum leptin and a reduction in muscle strength. Given the above relationships, we hypothesized that serum leptin could be associated with gait stability. To test this, 146 apparently healthy university students were recruited. Data collection involved anthropometric measurements, physical activity (PA) data, gait parameters, and serum leptin levels. A gait instability index was derived from the percentages of double support time and walking asymmetry (WA) collected from smartphones. Females demonstrated higher leptin levels and WA despite a lower body mass index (BMI). Lower PA levels were also observed among females. Leptin levels were negatively correlated with WA, step count, and vigorous PA (*p* < 0.05). These correlations remained significant following correction for leptin by BMI. Using logistic regression, a higher leptin-to-BMI ratio was associated with high gait instability (OR = 9.97, 95%CI: 4.17−23.84, *p* < 0.001). After stratification by sex, this association was only evident among females (OR = 6.09, 95%CI: 1.04−35.56, *p* = 0.045). These findings suggest a sex-based association between serum leptin and gait stability among apparently healthy students.

## 1. Introduction

Gait stability is the ability of the body to maintain balance and control and stay steady while performing activities like walking or running [1]. It reflects the intricate coordination of various components of the nervous and muscular systems [2]. Gait stability maintains walking/running steadiness by counteracting the effects of several factors, intrinsic or extrinsic, that disturb the neuromuscular balance [3]. Examples of intrinsic factors include aging and chronic diseases like diabetes mellitus, Parkinson’s, or Alzheimer’s [4]. These diseases and/or their complications negatively affect gait stability by inflicting a deterioration in muscle mass and strength or by altering signaling at the neuromuscular junction [4,5]. Extrinsic environmental factors include rugged carpets, wet floors, or unsafe footwear [6].

Falls are occurrences in which an individual unwillingly comes to rest on the ground or floor [7]. Fall-related injuries represent a growing public health problem [8]. For example, figures reported by the World Health Organization (WHO) estimated that fall-related fatalities rank as the second most common cause of deaths related to unintentional injury and are second only to road traffic accidents [8].

Non-fatal falls also represent a burden on the health sector. Worldwide estimates in 2021 indicated that annually 37.3 million falls have sufficient repercussions to demand medical attention [9]. The epidemiology of fall-related fatalities shows a clear age disparity with individuals older than 60 years having the highest death rates regardless of the region of the world under investigation [9].

Gait stability is a measurable factor with documented effects on the risk of falling [3,10]. The current consensus indicates that a reduction in gait stability increases the risk of falling and fall-related injuries [10]. It is thus not surprising that several reports have shown that a reduction in gait stability is a significant risk factor for morbidity and/or mortality [11,12].

A gradual decline in gait speed and step length, two parameters that reflect gait stability, are considered part of the normal aging process [13]. Regardless of the age-related decline, better gait stability remains a target of the elderly population, especially since current research has demonstrated that better gait stability is associated with better survival and better health outcomes [14]. Based on the above discussion, it is of interest to public health systems to understand the factors that modify gait stability. This would inform the discovery of novel strategies that improve gait stability and could thus reduce the risk of falling. This conundrum represents one of the major aims of this report.

Spatiotemporal gait parameters are considered a useful marker for assessing gait stability and gait health [15]. Some examples of spatial parameters are the step length, step width, and stride length. On the other hand, the number of steps/minute (cadence), gait speed, and the time and duration of gait cycle phases are considered temporal gait parameters [16]. Assessment of these parameters usually involves specialized systems and equipment and is only performed in specialized centers. Such systems include gait mats or walkways, motion capture cameras, wearable devices that include sensors, and electromyography [16].

Apple Inc. has introduced a new feature to their smart devices, which allows iPhone/iWatch users to monitor several spatiotemporal gait parameters (collectively referred to in the application as measures of walking stability). This feature uses built-in sensors that automatically track gait parameters without requiring conscious measurement initiation [17,18]. This offers the advantage of subconsciously estimating gait stability without an obligatory visit to specialized health centers. Interestingly, it was recently reported that estimating gait stability via the Apple Health Application strongly correlated with the results obtained using the classical approaches described above [17].

Leptin, a peptide hormone synthesized by the adipose tissue, plays an integral role in the regulation of energy expenditure, metabolism, and appetite [19]. Leptin is increasingly recognized for a plethora of physiological functions that extend beyond its classically described role in mediating energy hemostasis and controlling appetite [20]. As of late, leptin has stood out as one of the key mediators of the inflammatory response in different systems including the reproductive and musculoskeletal systems [20,21]. The pro-inflammatory properties of leptin may partially explain the association of serum leptin levels with multiple chronic diseases [21,22,23,24,25].

The interest of the scientific community in studying the pathobiology of leptin was first triggered by observational and mechanistic studies reporting an association between leptin levels with adiposity and other obesity-related conditions [26]. However, evidence accumulating over the years has consistently demonstrated a wider role for leptin in other disease categories including autoimmune and neurodegenerative diseases, and cancer biology [21,27]. As explained above, the role of leptin in the pathophysiology of these diseases could be related to its role in mediating inflammation.

An intriguing facet of leptin biology includes its association with muscle mass/strength. A number of reports showed that elevated levels of serum leptin and other inflammatory cytokines were linked with lower skeletal muscle mass and a higher risk of sarcopenia [28,29]. Sarcopenia is a disease which predominantly affects the elderly [30] and is characterized by the continuous loss of muscle strength and function [30].

Higher muscle strength is usually a consequence of increased muscle mass. This relationship may explain why leptin, in addition to its association with muscle mass, was also found to be negatively associated with muscle strength [31,32] and with the risk of sarcopenia [32].

Muscle mass/strength is one of the major determinants of gait stability. Unsurprisingly, higher levels of serum leptin were associated with a progressive decrease in gait speed in elderly females [33]. This is supported by observations made by Kao et al. In their study, it was reported that a negative association existed between leptin and gait speed in both sexes [31]. In another study which included 1573 individuals older than 60 years, Lana et al. found that higher leptin levels were associated with increased muscle weakness and increased risk of exhaustion among participants independent of body fat percentage [34].

In general, females have a higher fat percentage than males of the same height and weight [35]. The adipose tissues of females also have a higher rate of leptin secretion than males [36]. Taken together, this may explain why the levels of serum leptin are generally higher in females than their male counterparts [36,37].

It is interesting to note that data from several studies suggested that females generally have a higher risk of falling than males [38,39]. Indeed, the female sex per se is considered a risk factor for a reduction in gait stability [40]. Considering the relationship between higher levels of serum leptin with reduced muscle strength/mass, it is conceivable that sex-based differences in gait stability could be partially explained by the established differences in serum leptin levels between the two sexes. This, however, remains to be fully elucidated.

The purpose of this report was to determine the relationship between several parameters of gait stability and physical activity (PA) with the serum levels of leptin in a cross-section of apparently healthy young adults and to explore if sex-based differences existed in these associations.

## 2. Materials and Methods

### 2.1. Design, Participants, and Data Collection

Data were collected through a cross-sectional survey of 146 university students at Jordan University of Science and Technology (JUST). Study participants were students between 18 and 40 years of age of both sexes and of Jordanian descent. Only students who carried an iPhone with an active health application for at least 12 full months to the day of data collection were eligible to participate in the study. The above application, if active, can track several PA and gait stability parameters of the user [17,18].

Students with chronic or neurological diseases/disorders such as diabetes mellitus, hypertension, polycystic ovarian syndrome, multiple sclerosis, or any endocrine-related disorder were excluded. Students with any physical disability and those who reported that they only intermittently carry their iPhones were also excluded. Due to the potential influence of footwear on gait parameters, female participants were asked about their typical footwear choices. Those who reported wearing heels on a daily basis or for more than two days per month (for over three hours on each day) were excluded.

All procedures were approved by the Institutional Review Board (IRB) at Jordan University of Science and Technology (Protocol ID: 15/160/2023 date: 11 May 2023). A research coordinator explained the study goals prior to data collection. Written informed consent was obtained from all enrolled participants.

Data were collected during the period from May to August of 2023. The sampling was clustered to ensure the representation of both undergraduate and master’s students at JUST. Data collection was performed at different sites within JUST and included students enrolled in all programs (undergraduate or master) offered at JUST. Data collection took place at different times of the day between 8 am and 4 pm. This approach aimed to minimize the potential bias associated with targeting specific groups of students belonging to any single study discipline.

### 2.2. Measurements

#### 2.2.1. Anthropometric Measurements

Height and weight were self-reported by the participants. Body mass index (BMI) was calculated by dividing weight in kilograms (kgs) by height in centimeters squared (cm^2^) (BMI = kg/m^2^). Waist circumference (WC) was measured while participants stood upright. WC measurements were performed as described by Alfaqih et al. [41]. Hip circumference (HC) was measured at the level of the greater trochanters in the horizontal plane (parallel to the floor). WC and HC measurements were performed using a flexible non-stretchable measuring tape and were recorded to the closest 0.5 cm. The waist-to-hip ratio (WHR) was calculated using the following formulae (WHR= WC/HC).

#### 2.2.2. Physical Activity

Two approaches were used to assess the PA of the study participants. The first approach was objective and employed one of the features of the health application stored on the iPhone carried by the participants. Specifically, the above health application has a feature that calculates the number of steps averaged over a specific period of time. Herein, step count was used as a surrogate variable for PA. Data extracted from the application included daily step averages over the last six months or year (used in the final analysis as two separate variables).

Second, a standardized subjective approach was employed. This approach required that all recruited participants fill out a PA questionnaire for the subjective assessment of PA. For this purpose, the Global Physical Activity Questionnaire (GPAQ) was used. The GPAQ is a tool developed by the World Health Organization (WHO) for PA surveillance. The validated Arabic version (GPAQ-A) was used in this report [42].

Data collected from the GPAQ-A were used to calculate metabolic equivalent task (MET) minutes [43]. The above questionnaire could be used to calculate the number of minutes a participant spends performing PA of moderate or vigorous intensity per week by listing an example of each type of PA. The questionnaire considers that PAs of moderate intensity are equivalent to 4 METs while PAs of vigorous intensity are equivalent to 8 METs. One MET is equivalent to the amount of energy used at rest (i.e., baseline) [43].

The questionnaire was also used to categorize participants into either physically active or inactive individuals. For this classification, a PA score was first calculated by calculating the sum of MET minutes per week related to PAs of both moderate and vigorous intensity.

Participants who achieved a PA score of 600 or above were considered physically active. On the other hand, participants who achieved a score of less than 600 were considered physically inactive. This classification matches the guidelines of the World Health Organization (WHO) and the recommendation of the current literature for the prevention of several chronic diseases (e.g., hypertension, diabetes, chronic neck pain, chronic back pain, depression, anxiety, and lung cancer) [43,44,45].

#### 2.2.3. Gait Parameters

Gait stability parameters were extracted from the participants’ smartphone data. iPhones have a feature that allows users to monitor their gait stability and coordination. This feature was initially introduced within the health application of the smartphone in the iPhone 8, iOS 15, and has remained operative in subsequent versions. The above feature of the smartphone employs custom algorithms that assess data obtained from motion sensors built into the iPhone. The smartphone automatically measures and records several spatiotemporal gait parameters [17,18], which include gait speed (km/hour), step length (cm), double support time (DST) percentage (%), and walking asymmetry (WA) percentage (%).

The DST refers to the percentage of time during a walking cycle in which both feet of an individual are touching the ground. In general terms, a lower DST percentage indicates that an individual is spending less time during their gait cycle on both feet, an activity that correlates with higher gait stability.

On the other hand, the WA refers to the percentage of time of a gait cycle in which the steps of one foot are different (i.e., asymmetric) from the steps of the other foot. A lower WA percentage generally correlates with a more stable gait [17,18].

#### 2.2.4. Serum Leptin

A total of 1 ml of whole blood was collected from each participant by a certified phlebotomist. Collections were performed in a plain tube with a gel clot activator (AFCO, Amman, Jordan). To recover the serum, blood samples were centrifuged at 4500 rpm for 5 min at room temperature. Collected serum was then distributed into six 1.5 mL centrifuge tubes. The tubes were frozen in liquid nitrogen and then stored at −80 °C for later use.

Quantitative measurements of serum leptin were described previously [23,24]. In brief, levels of serum leptin were quantitated using a sandwich enzyme-linked immunosorbent assay (ELISA) system (Human Leptin DuoSet^®^, Cat. no. DY398-05, R&D systems Inc., Minneapolis, MN, USA). The optical density (OD) of each well was measured using the ELx800 microplate reader (BioTek Instruments, Inc., Winooski, VT, USA) set to a wavelength of 450 nm.

It is well established that serum leptin levels are positively associated with increasing adipose tissue mass. In order to account for the above relationship, leptin levels were adjusted to BMI (a surrogate of adipose tissue mass) via the calculation of a leptin-to-BMI ratio (leptin/BMI). This calculation was performed for each participant by dividing the serum leptin concentration (ng/mL) by the BMI (kg/m^2^) of the participant.

### 2.3. Statistical Analysis

Statistical analysis was performed using the Statistical Package for Social Studies (SPSS) software (version 27, IBM, Armonk, NY, USA). Categorical data were presented as frequency (percentage) (n (%)) while continuous data were presented as mean (±standard deviation (SD)).

Descriptive statistics were calculated for the study participants, including age, sex, and level of education. An independent samples *t*-test was used to compare anthropometric measurements (weight, height, BMI, WC, HC, and WHR), leptin, leptin/BMI, and gait parameters (step length, walking speed, DST percentage, WA percentage, step count, and MET minutes) between males and females. An independent samples *t*-test was also used to compare leptin/BMI, vigorous MET minutes, and step count between participants with high vs. low instability.

Pearson’s chi-squared test was used to assess the association between sex and physical inactivity (per WHO recommendations). Pearson’s correlation test was used to assess the strength of the association between leptin and anthropometric measurements (BMI, WC, HC, or WHR), MET minutes, step count, or gait parameters (step length, gait speed, DST percentage, and WA percentage).

A gait instability index was calculated for each participant based on the one-year averages of DST and WA percentages. The following criteria were used in the calculation. In brief, the mean value of the DST or WA percentages was first calculated. Participants who had a DST or WA percentage higher than the mean value of that specific variable were categorized as having high instability. On the other hand, participants who had a DST and a WA percentage that were both below the mean of each parameter were categorized as having low gait instability.

Binary logistic regression analysis was then used to examine the relationship between the gait instability index calculated above and leptin/BMI following adjustment for PA levels. In this analysis, leptin/BMI was also used as a binary variable (high vs. low) with the mean being the cut-off value. The level of statistical significance for all analyses was set to 0.05.

## 3. Results

### 3.1. Baseline Characteristics, Anthropometric Measurements, and Leptin Levels

The characteristics of the participants are described in Table 1; the mean age of the population (n  =  146) was 24.15 ± 4.56 years. The majority of the participants were female (65.10%). Approximately half of the population (50.7%) were still pursuing a bachelor’s degree (undergraduates) at the time of data collection, while the other half (49.3%) were master’s students.

One of the major goals of this report was to validate previous data showing that there are sex-based differences in serum leptin levels and determine if these differences are independent of adipose tissue mass.

To achieve the above goal and adjust for adipose tissue mass, we first compared the levels of several anthropometric measurements between males and females in the population. The results of this analysis are shown in Table 2. Males in the population had significantly higher BMI, WC, HC, and WHR (*p* < 0.05) (Table 2).

**Figure 1:**
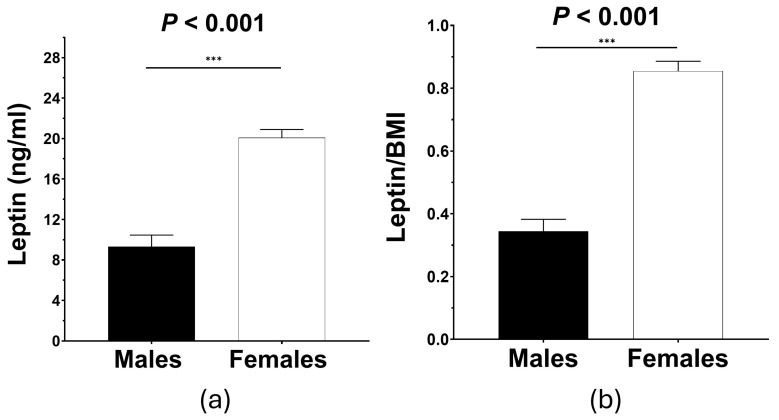
Sex-based differences in serum leptin. Comparison of (**a**) leptin (ng/mL) and (**b**) leptin/BMI between males vs. females showed significantly higher leptin or leptin/BMI in females. BMI: body mass index. *** denotes *p* < 0.001, by the independent samples *t*-test. Bar graphs present the mean (SEM). n_males_ = 51, n_females_ = 95.

Following the above analysis, serum leptin levels (separately or following adjustment with BMI) were compared between males vs. females in the population (Figure 1). Herein, it was demonstrated that females had 2.6-fold-greater serum leptin levels compared to males (*p* < 0.001) and around 3-fold-greater serum leptin levels following correction for BMI (a surrogate of adipose tissue mass) (*p* < 0.001).

As shown in Table 3, it is interesting to note that leptin levels showed a statistically significant positive correlation with BMI, WC, HC, and WHR in males in the population. Females showed similar relationships; however, leptin levels were not significantly correlated with the WHR (*p* > 0.05).

### 3.2. Physical Activity and Gait Parameters

Following our observations above, which validated the previous literature that showed sex-based differences in serum leptin levels, the authors sought to determine if there were any sex-based differences in PA or gait parameters in the population under study.

Two methods were used for the assessment of PA: an objective method based on data collected from the health application of the participants’ smartphones and a subjective method based on a standardized assessment of PA using the GPAQ.

**Figure 2:**
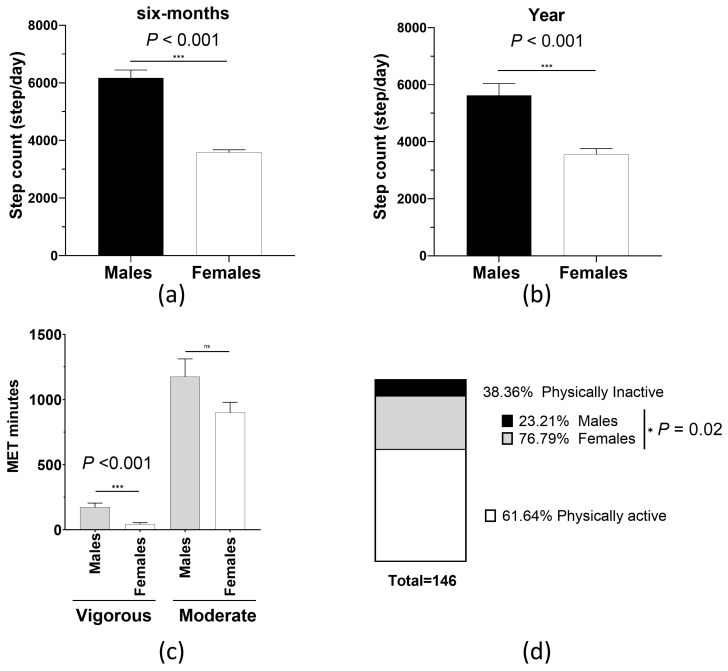
The association between sex and physical activity. The comparison of (**a**) six-month or (**b**) one-year daily average step count between males vs. females showed a significantly higher step count per day in males. (**c**) Males spend significantly more MET minutes performing vigorous-intensity physical activity than females. (**d**) Females represent a significantly larger percentage of the participants classified as physically inactive according to WHO recommendations. Physically inactive subjects are those who perform < 600 MET minutes/week. MET: metabolic equivalent task * *p* < 0.05, *** *p* < 0.001. ns, not significant. Bar graphs present the mean (SEM). n_males_ = 51, n_females_ = 95.

The results of the objective assessment of PA performed by extracting average step count data over the last six months or year are shown in Figure 2. This analysis showed that males had a significantly higher daily average step count than females over the past six months (Figure 2a) or year (Figure 2b) from the time of data collection.

Interestingly, a similar trend was observed upon subjective assessment of PA using the GPAQ. In this approach, it was demonstrated that males reported significantly higher MET minutes performing PA of vigorous intensity (Figure 2c). Males reported spending more time performing PA of moderate intensity; however, the difference did not reach statistical significance (Figure 2c).

According to the WHO, the minimum recommended PA level is 600 MET minutes per week [43]. The above cut-off was used to classify the study participants into physically active or inactive individuals. The authors then compared the percentage of individuals who fell under either classification in males vs. females. The findings of this comparison are shown in Figure 2d. First, it was revealed that more than one-third of the population (38.36%) did not meet the minimum recommended PA level according to WHO standards (i.e., ≥600 MET minutes per week). Notably, a significantly higher proportion of females (76.79%) fell into this category compared to males (23.21%), with a *p* value of 0.02 (Figure 2d).

In light of the findings above showing sex-based differences in leptin and PA, it was of interest to determine if significant differences existed in gait stability. Several gait stability parameters were collected from the smartphone data of the participants. These parameters included step length (cm), gait speed (km/hour), the percentage of DST, and the percentage of WA. Data included average values collected over the last six months or one year.

A comparison of the above parameters between males vs. females in the population is shown in Table 4. The gait parameter analysis described above revealed the absence of any sex-based differences in all of the parameters examined except for a significantly slower walking speed among females over the past six months (*p* = 0.045) and a higher percentage of WA among females over the last six months or one year (*p* < 0.001). This finding suggested a sex-specific difference in gait stability with lower gait stability among females compared to males in the population.

### 3.3. The Association Between Leptin, Physical Activity, and Gait Parameters

As mentioned above, significant differences existed in serum leptin, PA, or WA (a gait parameter) between males vs. females in the population. The authors next wanted to test if there were any significant correlations between leptin (corrected for BMI) and PA or gait stability. Moreover, it was also of interest to determine if there were any sex-based associations between serum leptin and gait stability.

To achieve the purposes above, several analyses were performed. Using bivariate correlations (Table 5), it was observed that the leptin/BMI ratio was negatively correlated with vigorous MET minutes, six-month average step count, one-year average step count, six-month average WA percentage, and one-year average WA percentage (*p* < 0.05).

Since WA percentage only represents one facet of gait stability, a more comprehensive binary measure of gait stability was devised. The measure was called the gait instability index and was derived from the DST and WA percentages (see Section 2). According to the above measure, the participants were categorized as either having high or low gait instability.

**Figure 3:**
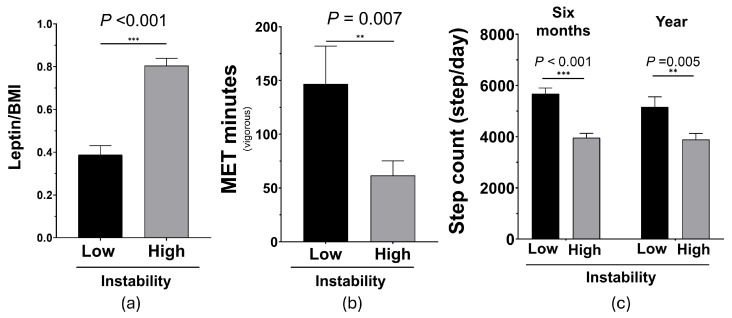
The association between gait stability, serum leptin, and parameters of physical activity. High instability was significantly associated with (**a**) higher leptin/BMI, (**b**) lower vigorous MET minutes, and (**c**) lower step count. BMI: body mass index; MET: metabolic equivalent task; ns: not significant. ** *p* < 0.01, *** *p* < 0.001, by the independent samples *t*-test. Bar graphs present the mean (SEM). n_lower_ = 45, n_higher_ = 101.

Next, it was demonstrated that individuals with high gait instability (according to the derived index described above) also had a significantly higher leptin/BMI ratio (Figure 3a). This result was consistent with the results of the bivariate correlation between leptin/BMI and WA percentage described above. Additionally, gait stability was also associated with PA as shown in Figure 3b,c. Specifically, high gait instability was significantly associated with lower vigorous MET minutes (Figure 3b) (*p* < 0.05) and lower average step count (six-month or one-year average) (Figure 3c) (*p* < 0.05).

One of the major aims of this report was to examine if a sex-based association existed between serum leptin and gait stability. Therefore, a logistic regression model was used with the gait instability index as a binary outcome variable. The leptin/BMI ratio was used as a predictor binary variable (high vs. low). The model was run on the entire population and then it was performed following stratification of the population into males or females. In this analysis, shown in Table 6, the leptin/BMI ratio significantly increased the risk of gait instability in the total population (OR = 9.97, 95% CI: 4.17–23.84, *p* < 0.001) and in females (OR = 6.09, 95% CI: 1.04–35.56, *p* = 0.045) but not in males (*p* = 0.92). It was concluded from the above set of analyses that sex-based differences in serum leptin may partially explain lower gait stability in females.

## 4. Discussion

A stable gait is defined as a body stance that prevents falls and subsequent injury [3]. Despite being an innate developmental phenotype of bipedal (humans as an example) or quadrupedal animals, maintenance of a stable gait is a complex process and requires intricate coordination of visual and proprioceptive feedback with the neuro-muscular control of body position [2,3]. Factors, intrinsic or extrinsic, may disrupt the above complex interaction between input signals with the neuronal control of limb movements [3], leading to lower gait stability.

Lower gait stability is closely associated with fall risk. Fall-related injuries are a major source of morbidity and mortality, especially in the elderly [46], and represent a financial burden on the health sector [46]. In view of the magnitude of this problem, it is imperative to identify modifiable factors that affect the risk of falling.

Considering that factors that modify gait stability would presumably modify the risk of falling, one of the goals of this report was to test the association of leptin hormone with gait stability. Highlighting such an association would set the stage for future trials that attempt to modify gait stability via the modification of serum leptin levels.

Leptin hormone levels show sex-based differences and are generally higher in females independent of differences in adipose tissue mass [37,47]. Considering that the female sex per se appears to be associated with lower gait stability, the second goal of this report was to assess if the lower gait stability in females is a reflection of a sex-based association of gait stability with leptin hormone levels. To avoid any potential bias from the presence of chronic diseases known to affect gait stability, this report investigated the above associations in a cross-section of apparently healthy university students.

In this section of the report, we will first discuss the significance of discovering a potential axis between higher leptin levels with lower gait stability in the management of gait-related problems. We will also provide a tentative molecular mechanism that explains how leptin may be affecting gait stability.

The discovery of a correlational axis linking an elevation in leptin hormone levels with lower gait stability highlights several approaches that could be used to enhance gait stability by reducing the serum levels of leptin. One such approach could be lifestyle interventions accompanied by dietary modifications. For example, the ACOORH trial (Almased Concept against Overweight and Obesity and Related Health Risk) was a multicenter, randomized trial performed on obese individuals [48,49]. One of the arms of this trial included lifestyle modifications accompanied by a meal replacement plan that included low-carbohydrate, protein-rich meals [50]. Serum leptin measurements found that meal replacement significantly reduced leptin levels compared to lifestyle modifications alone (i.e., the other arm of the study). The implementation of comparable interventional strategies with gait parameters as one of the endpoint measurements is a future direction of the research team and would highlight how a reduction in serum leptin may lead to better gait stability.

The exact mechanism by which higher levels of serum leptin would contribute to lower gait stability is still not clear. One tentative mechanism that may explain the link between leptin and gait stability relies on the relationship between leptin and muscle mass/strength [28,29].

It is becoming more evident that the adipose tissue itself, via its endocrine function, plays a role in reducing skeletal muscle strength apart from the fat-to-muscle ratio. For example, in sarcopenia, it was found that the reduction in muscle mass was associated with an increase in the secretion of IL-6, TNF-α, and IL-1β by the intramuscular adipose tissue [51]. Intriguingly, in this context, it was observed that leptin was secreted by adipose tissues and caused an upregulation in the secretion of IL-6 [52]. Hyperleptinemia is known to be associated with systemic inflammation [53]. This presents one mechanism by which leptin may contribute to muscle health and gait stability.

In addition to the role of leptin as a pro-inflammatory adipokine, there are other mechanisms by which leptin may regulate skeletal muscle homeostasis. For example [54], Baldwin et al. found that leptin reduced the expression of myosin heavy chain I. The above protein is abundant in type I fibers of skeletal muscles [55]. These fibers are described as slow twitch endurance fibers which depend on oxidative phosphorylation as a major source for their contractile energy [56]. Leptin was also found to affect skeletal muscle angiogenesis [57]. Given that skeletal muscle strength and mass are major determinants of posture and gait stability [58], it is plausible that leptin via its multifaceted roles in muscle physiology could play a role in gait stability. 

Based on the above, an endocrine loop could be envisioned in which a change in the secretory profile of the intramuscular adipose tissue could cause an increase in leptin hormone secretion, a process that would instigate an increase in the secretion of IL-6 and other pro-inflammatory cytokines. An increase in the concentration of these cytokines beyond certain threshold levels may stimulate the damage of myocytes, causing an eventual reduction in muscle strength [52]. This by itself would result in a decrease in gait stability. The damage of myocytes could also be induced by an increase in intracellular oxidative stress [59], yet another feature known to be associated with an increase in serum leptin levels [59].

PA directly affects muscle mass and neuromuscular coordination [60]. It is thus not surprising that PA is yet another modifiable factor that may impact gait stability. Females have a universally lower tendency to engage in PA [61,62]. Lower PA in females (including the females of this population) may also be another factor that may explain the sex-based differences observed in gait stability. Although bivariate analysis between the devised instability index (high vs. low) and PA supported the presence of a relationship between PA and gait stability. The multivariate regression model listed in this report did not support such a relationship. This, however, may be a reflection of the small size of the population under study in this report.

A key strength of this investigation stems from the novelty of its findings. Indeed, the work presented in this report offers valuable insights into a tentative physiological axis that connects leptin hormone metabolism with gait stability. Although preliminary and correlational in its nature, this report appears to be one of the first to establish an association between higher levels of serum leptin and a reduction in gait stability.

Another area of strength lies in the relative novelty of the methodology used to assess gait parameters and PA. The method of assessment used in this report, which depends on data extracted from the smartphone health application, offers several advantages over the conventional tools and methods classically used to assess gait parameters.

Smartphones are popular and accessible and provide real-time data registered without the conscious knowledge and awareness of the user [17,18]. Moreover, our assessment of PA used both subjective and objective methods with compatible findings between the two assessment methods. This by itself served as an internal control that validated the findings concluded from smartphone data and added strength to the design of this study.

In view of a number of limitations and despite the strengths mentioned above, it is still premature to generalize the findings of this report. Firstly, this investigation is underpowered and limited by a relatively small sample size. A future study with a larger sample size is required to validate the results of this report.

Secondly, the relationships between most of the variables collected in this report including obesity, PA, gait stability, and leptin are complex and subject to modification by many other confounders, such as the use of supplements and/or medications, sleep quality, and mental health status. Data describing these confounders were not collected, making it difficult to conclude a solid and evidence-based relationship between the study variables based only on correlational data.

Moreover, future studies are still required in order to decipher the mechanism underlying the association between leptin and gait stability. These studies remain a future direction of the research team and may help uncover molecular markers that could be targeted or monitored in interventional studies that aim to enhance gait stability.

Conducting the study on an apparently healthy and young population had the advantage of avoiding the confounding effects of age and chronic disease. However, this design limited the applicability of the findings to the apparently healthy population only. This makes it difficult to claim that the axis between leptin and gait stability is also found in the elderly and in patients with chronic disease. Validating the findings of this report in the elderly and in patients with chronic disease remains a future direction of the research team.

It is documented that carrying the smartphone closer to the center of mass of the individual (i.e., closer to the hip) results in more frequent measurements made by the built-in sensors of the smartphone. It thus could be conceived that smartphone-carrying habits of the participant could be another confounding factor that may affect the accuracy of the findings of this report.

Nonetheless, it is important to note that the sensors of the iPhone are programmed in such a way as to register a lower number of measurements in a sub-optimal carrying location over recording values of lower accuracy [18].

Indeed, the sensors were tested in settings of mixed iPhone-carrying locations by individuals. In these settings, the measurements recorded by the sensors demonstrated good validity, reliability, high positive predictive values, and low false-negative rates [18]. The above discussion supports the robustness of the findings of this report regardless of the potential confounding effect of the smartphone-carrying location.

## 5. Conclusions

In conclusion, the observational study described in this report uncovered significant associations between physical activity, gait stability, and leptin hormone serum levels. Sex-based stratification revealed that the association between serum leptin levels and gait stability is presumably stronger in females than males and is independent of BMI (a surrogate marker of adipose tissue mass).

Other important results include our finding that females tend to be less physically active compared to males, a factor that may also contribute to the observed differences in gait stability. Within the same lines above, it was discovered that males who exhibited higher PA demonstrated lower leptin levels and lower WA percentages despite higher BMI and WC. These findings emphasize the importance of maintaining the WHO-recommended levels of PA for overall better health, higher gait stability, and a lower risk of falling.

Moving forward, future investigations using larger sample sizes, longitudinal designs, or outcome-oriented interventional studies could be performed to establish causality and further elucidate the mechanistic role of leptin in gait stability. From a public health standpoint, this study highlights the urgent need for educational programs and initiatives specifically designed to target the student female population in Jordan in order to improve their PA levels. These programs will represent a primary prevention approach to mitigate the effects of lower gait stability in the young adult (or even older) populations.

## Figures and Tables

**Table 1:** General characteristics of the study participants.

Variable	n (%)
Sex Males Females	51 (34.90%) 95 (65.10%)
Age	24.15 ± 4.56
Educational level Undergraduates Master’s students	74 (50.68%) 72 (49.32%)
Total	146

Data are presented as mean ± standard deviation or n (%).

**Table 2:** Sex-based differences in anthropometric measurements.

Variable	Total N = 146	Males n =51	Females n =95	*p* Value
Weight (kg)	67.19 ± 13.92	78.41 ± 13.35	61.17 ± 9.95	<0.001
Height (m)	1.67 ± 0.09	1.76 ± 0.07	1.61 ± 0.05	<0.001
BMI (Kg/m^2^)	24.00 ± 3.89	25.21 ± 4.05	23.35 ± 3.65	0.005
WC (cm)	79.05 ± 12.70	87.55 ± 13.85	74.44 ± 9.26	<0.001
HC (cm)	98.72 ± 10.02	101.75 ± 9.45	97.09 ± 0.99	0.007
WHR	0.80 ± 0.08	0.86 ± 0.07	0.77 ± 0.06	<0.001

Data are presented as mean ± standard deviation. *p* values were calculated using an independent samples *t*-test. BMI: body mass index; WC: waist circumference; HC: hip circumference; WHR: waist-to-hip ratio.

**Table 3:** Correlation of leptin with physical activity or gait parameters.

	Leptin (ng/mL)	Males n = 51	Females n = 95
BMI (kg/m^2^)	r	0.68	0.44
	*p* value	<0.001	<0.001
WC (cm)	r	0.70	0.46
	*p* value	<0.001	<0.001
HC (cm)	r	0.71	0.44
	*p* value	<0.001	<0.001
WHR	r	0.52	0.17
	*p* value	<0.001	0.10

r represents Pearson’s correlation coefficient. BMI: body mass index; WC: waist circumference; HC: hip circumference; WHR: waist-to-hip ratio.

**Table 4:** Sex-based differences in gait parameters.

		Males n = 51	Females n = 95	*p* Value
Step length (cm)	six months	72.35 ± 10.09	72.03 ± 4.87	0.83
	year	73.08 ± 9.91	71.59 ± 6.09	0.26
walking speed (km/hour)	six months	4.56 ± 0.53	4.41 ± 0.39	0.045
	year	4.55 ± 0.54	4.51 ± 0.34	0.66
DST (%)	six months	28.15 ± 1.11	28.27 ± 1.36	0.62
	year	28.12 ± 1.31	28.20 ±1.31	0.71
WA (%)	six months	4.61 ± 3.02	6.65 ± 2.47	<0.001
	year	4.30 ± 2.30	6.15 ± 1.61	<0.001

Data are presented as mean ± standard deviation. *p* values were calculated using the independent samples *t*-test. DST, double support time; WA: walking asymmetry.

**Table 5:** Correlation of leptin with physical activity or gait parameters.

Variable		Leptin r (*p* Value)	Leptin/BMI r (*p* Value)
Vigorous MET minutes		−0.20 (0.02)	−0.23 (0.005)
Moderate MET minutes		−0.09 (0.26)	−0.11 (0.19)
Step count	six months	−0.35 (<0.001)	−0.43 (<0.001)
	year	−0.33 (<0.001)	−0.38 (<0.001)
Step length	six months	0.10 (0.23)	0.07 (0.41)
	year	0.01 (0.87)	0.00 (1.00)
Walking speed	six months	0.06 (0.46)	0.05 (0.51)
	year	0.13 (0.11)	0.14 (0.10)
DST	six months	0.11 (0.18)	0.07 (0.38)
	year	0.08 (0.33)	0.04 (0.62)
WA	six months	0.23 (0.006)	0.28 (<0.001)
	year	0.25 (0.002)	0.29(<0.001)

r represents Pearson’s correlation coefficient. DST: double support time; MET: metabolic equivalent task; WA: walking asymmetry.

**Table 6:** Binary logistic regression.

	Leptin/BMI (High Vs. Low)	
	OR (95% CI)	*p* Value
Total population (n = 146)	9.97 (4.17–23.84)	<0.001
Males (n = 51)	1.10 (0.19–6.33)	0.92
Females (n = 95)	6.09 (1.04–35.56)	0.045

Outcome variable: gait instability (binary variable). BMI: body mass index; CI: confidence interval; OR: odds ratio.

## Data Availability

The raw data supporting the conclusions of this article will be made available by the authors on request.
